# Urinary VP1 Flow Cytometry as a Complementary Approach for BK Polyomavirus Monitoring: A Proof-Of-Concept Study

**DOI:** 10.3389/ti.2026.15780

**Published:** 2026-04-08

**Authors:** Haris Omic, David Vecsei, Michael Eder, Karim Abd El-Ghany, Wolfgang Winnicki, Alice Schmidt, Sebastian Kapps, Daniela Gerges, Robert Strassl, Ludwig Wagner, Farsad Eskandary

**Affiliations:** 1 Department of Medicine III, Division of Nephrology and Dialysis, Medical University of Vienna, Vienna, Austria; 2 Department of Clinical Virology, Medical University of Vienna, Vienna, Austria

**Keywords:** BK polyomavirus–associated nephropathy, kidney transplantation, liquid biopsy, urinary VP1 flow cytometry, viral biomarkers

## Abstract

Polyomavirus nephropathy (BKPyVAN) is a major cause of allograft dysfunction after kidney transplantation (KTX). While plasma BKPyV-PCR is the diagnostic gold standard, it may not fully reflect tissue injury. We conducted a prospective observational proof-of-concept study in 30 KTX recipients with BKPyV reactivation (November 2022–February 2024); 21 underwent kidney biopsy, 11 were diagnosed with biopsy-proven (BP)-BKPyVAN. Urine samples were analyzed by flow cytometry to quantify the potential of VP1-positive reno-urinary epithelial cells as a novel non-invasive marker of active tubular damage. The control cohort included 21 virology-negative patients. Median urinary VP1-positivity was higher in BP-BKPyVAN (33%, IQR 27–46) vs. non-BKPyVAN patients (5%, IQR 1–13; *p* < 0.001). The assay achieved an AUC of 0.98 (95% CI 0.93–1.00, p = 0.0003; cut-off: 11.7%; sensitivity = 91%, specificity = 89%) for BP-BKPyVAN. Longitudinally, median VP1-burden declined from 13% (IQR 4–29) at baseline to 0% (IQR 0–0.4). BKPyV-DNAemia declined rapidly, but plateaued at ∼4 × 10^2^–7 × 10^2^ copies/mL, whereas urinary VP1-positive cells became undetectable. Our preliminary results suggest that combining urinary VP1-positivity with plasma BKPyV-PCR may help distinguish BP-BKPyVAN from non-BKPyVAN within a BKPyV-reactivation cohort. Longitudinal VP1 tracking may indicate resolution of viral infection earlier than DNAemia. These findings are hypothesis-generating and require validation in larger independent cohorts.

## Graphical Abstract

**Figure:**
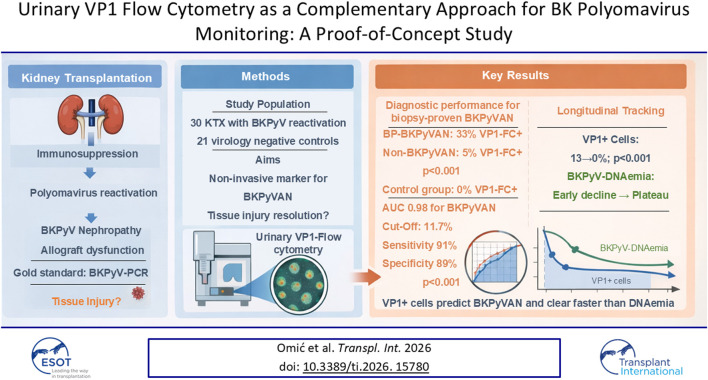

## Introduction

BK polyomavirus (BKPyV) is a highly prevalent human-specific pathogen residing in urogenital cells without pathogenicity in immunocompetent individuals [1–4]. However, its tropism and pathogenicity towards reno-urinary cells may change due to immunosuppression after kidney or stem cell transplantation [5, 6]. BKPyV may undergo a replicative surge, leading to lysis of tubular epithelial cells [6–8]. Infected cells are shed from the basement-membrane and can appear in urine as decoy cells [9, 10]. Despite the presence of BKPyV-DNAemia and high-level BKPyV shedding in the urine, affected patients typically do not exhibit systemic symptoms aside from kidney injury [11–13]. Physicians usually monitor BKPyV-replication after KTX to detect uncontrolled replication potentially leading to BK polyomavirus-associated nephropathy (BKPyVAN) [14, 15]. The gold standard for monitoring BKPyV uses quantitative polymerase chain reaction (qPCR) in plasma to assess BKPyV-DNAemia. In some centers urine sediment may also be assessed for the presence of decoy cells [16, 17]. In cases of worsening graft function, diagnostic proof of BKPyVAN requires KTX-biopsy, with evaluation of typical signs of lytic infection as well as immunohistochemical detection of simian virus 40 large T-antigen (SV40) in kidney cells [11, 18–21]. However, it has been observed that approximately 20% of BKPyVAN cases are still undetected using this method [13]. Consequently, alternative approaches such as qPCR for detecting BKPyV in urine and its sediment and the BK Haufen test were introduced to enhance sensitivity in detection strategies but are not part of routine care [22–24].

In this study, morning urine samples from KTX recipients with incipient or ongoing BKPyV-DNAemia and concomitant adaptation of immunosuppressive medication were collected longitudinally. Urine sediment cells were fixed, permeabilized, and stained via immunofluorescence by a novel BKPyV-specific capsid protein VP1 staining method to detect viral cellular presence, thereby allowing for a quantitative readout by flow cytometry (urinary-VP1-FC). This proof-of-concept study aims to evaluate a novel non-invasive FC-based immunofluorescence approach from urine sediment to enhance sensitivity for the success of ongoing viral clearance after the reduction of immunosuppression (IS). We investigated whether our biologically-sound approach may complement plasma PCR with its known lag time of viral clearance.

## Materials and Methods

### Patient Selection

**FIGURE 1:**
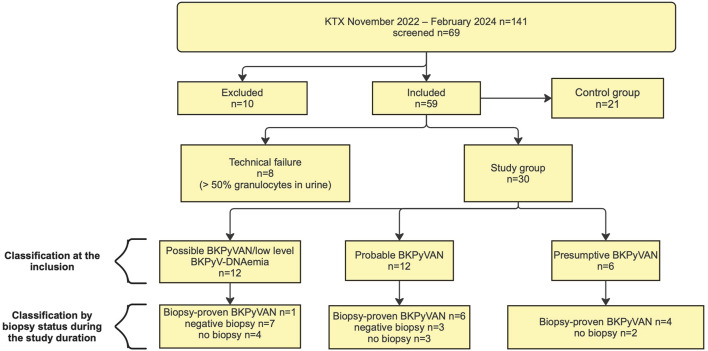
Study Flowchart. Ten patients did not meet the inclusion criteria and were therefore excluded from flow cytometry analysis due to prolonged urine storage time beyond the predefined limit (n = 3) or missing essential clinical data (n = 7). Classification according to BKPyV DNAemia was based on plasma BKPyV DNAemia levels at study inclusion, whereas classification by biopsy status was based on biopsy results obtained within the study period.

All KTX recipients (>18 years) at the outpatient clinic of the Department of Nephrology and Dialysis at the Medical University of Vienna between 11/2022 and 02/2024 were eligible for inclusion if they met one of the following criteria: Detection of BKPyV and/or JC polyomavirus (JCPyV) viremia on routine PCR; identification of decoy cells in routine urine sediment; a urinary viral load of BKPyV and/or JCPyV >10^8^ U/mL; biopsy-proven BKPyVAN (BP-BKPyVAN), which was performed in line with current consensus recommendations in the context of allograft dysfunction [11]. Patients were included at the time of clinically relevant BKPyV reactivation prompting diagnostic evaluation, rather than at a predefined virological stage, resulting in variable timing relative to transplantation and viral kinetics. In addition, a control group of clinically stable KTX patients without evidence of relevant BKPyV/JCPyV replication was included. Controls had no detectable DNAemia and no clinically relevant DNAuria according to current consensus definitions (urinary DNA <10^7^ copies/mL). The study flowchart is presented in Figure 1.

### Test Methods for Urine and Plasma BKPyV

Urinary and plasma BKPyV and JCPyV-DNA were quantified as part of routine post-transplant surveillance at our center in all kidney transplant recipients included in the study, following international screening recommendations, acknowledging that PyVAN may rarely be caused by JCPyV (for details, see Supplementary Materials) [25, 26].

### Definition of BKPyV-Associated Endpoints

BKPyVAN was categorized according to the most recent international consensus framework into four grades: possible, probable, presumptive, and definite BKPyVAN [13] (see details for each diagnostic tier in the Supplementary Materials).

### Flow-Cytometric Analysis of VP1-Positive Urinary Epithelial Cells

Urine cell pellets were ethanol-fixed and immunolabeled for BKPyV capsid protein VP1, with DAPI nuclear counterstain, and acquired on a FACSCanto flow cytometer [16]. Flow-cytometry data were processed using custom Python scripts in JupyterLab. Samples with marked granulocyturia were pre-specified as technical exclusions. The detailed protocol is provided in the Supplementary Materials.

### Cytopreparations

Urine sediment cells were maintained in culture medium as described above (RPMI 1640 supplemented with 2% FCS and ampicillin-streptomycin). A 60 μL aliquot was applied to the funnel of a cytocentrifuge and centrifuged at 1,200 rpm for 3 min. The resulting cytopreparation was air-dried for 1 h, followed by fixation in acetone for immunostaining or in the fixation solution of the Hemacolor^Ⓡ^ (Merck, Merck KGaA, Darmstadt, Germany; Millipore, SIG 1116740001) three-component kit for 4 min (see Supplementary Materials).

### Immunofluorescence of Urinary Cells on Cytopreparations

Cytopreparations were acetone-fixed and immunostained for BKPyV VP1 using either a mouse monoclonal primary (Invitrogen 4942, 1:60) or a rabbit anti-VP1 primary (1:1,000) on separate slides. After incubation (overnight at 4 °C or 2 h at room temperature) and PBS washes, Alexa Fluor 594 goat anti-mouse or Alexa Fluor 488 goat anti-rabbit secondary antibodies (1:700) were applied with DAPI nuclear counterstain. Slides were mounted in Vectashield and imaged on a Zeiss inverted confocal microscope; images were post-processed in Adobe Photoshop. Full step-by-step details are provided in the Supplementary Materials.

### Decoy Cell Staining

One cytopreparation was sequentially stained with eosin, followed by hematoxylin and a washing buffer, using the Hemacolor^Ⓡ^ staining kit (Merck, Merck KGaA, Darmstadt, Germany; Millipore, SIG 1116740001). Cell enumeration was performed via light microscopy (see Supplementary Materials).

### Statistical Analysis

Continuous variables were assessed for normality with the Shapiro-Wilk test and are presented as means ± standard deviations (SD) when normally distributed or as medians with interquartile ranges (IQR) for skewed data. Group comparisons were performed using the student’s t-test or the Mann-Whitney U test. Kruskal-Wallis’s test was used to compare the differences in BKPyV-DNAemia and urinary-VP1-FC counts in more than two groups. Complete statistical analysis is described in Supplementary Materials.

## Results

### Study Cohort

Between November 1st^,^ 2022 and February 29th^,^ 2024, 30 KTX recipients meeting our inclusion criteria for the study group and 21 KTX for the control group were analyzed. A flowchart of patient inclusion and exclusion is depicted in Figure 1. Table 1 provides all relevant patient dispositions and transplant-specific baseline characteristics. Median age was 59 years (IQR 49.8–64.0) and 19 (63%) recipients were male. Donor-specific antibodies were detected in one (3%) patient. The median HLA A/B/DR mismatch was three (IQR 2–4). As induction therapy the majority (97%) received IL-2 antibody basiliximab (20 mg on days 0 + 4). Maintenance immunosuppression consisted of tacrolimus/MMF/steroids in all patients (Table 1). The first flow‐cytometry assay was performed at a median of 11.5 months after KTX (IQR 2.7–18.1). At baseline, BKPyV-DNA was detectable at high levels in urine (median 3.3 × 10^6^ c/mL, IQR 2.5 × 10^1^–4.0 × 10^9^) and well detectable in plasma (median 4.6 × 10^3^ c/mL, IQR 2.5 × 10^2^–8.8 × 10^3^). Tacrolimus trough levels at the time of the first urinary VP1-FC sample (baseline) were at a median of 7.8 ng/mL (IQR 6.9–9.3), which was followed by a constant reduction due to significant BKPyV infection. In 29 (96%) patients the MMF dose was either reduced or MMF was paused. Serum creatinine mirrored this, with a median of 1.86 mg/dL (IQR 1.09–4.82) at baseline and a subsequent increase from months one to three, which was afterwards followed by a stabilization phase (Table 1; Supplementary Table S1). Renal allograft biopsy was performed in 21 of 30 patients (70%) with 10 being classified as biopsy-negative and 11 as biopsy-proven BKPyVAN. With respect to the timing of the biopsy, eight (38%) were performed at a median of 1 month (IQR 1.0–2.0) before the first urinary VP1-FC assessment - of which none showed signs of BKPyVAN - and 13 biopsies (62%) were performed at a median of 1 month after urinary VP1-FC (IQR 0.0–4.0, Supplementary Figure S1). Protocol biopsies accounted for 40% (4/10) of the biopsy-negative group and 18% (2/11) of the biopsy-proven group (p = 0.36). Three of the eight biopsies performed prior to VP1 sampling were protocol biopsies.

**TABLE 1:** Patient disposition and baseline characteristics.

Variable		
Age, years (median, IQR)	59	50–64
Recipient male sex (n, %)	19	63
Donor male sex (n, %)	12	55
Cause of ESRD		
Congenital* (n, %)	10	33
Glomerular** (n, %)	4	13
Metabolic/vascular*** (n, %)	11	37
Unknown etiology (n, %)	4	13
Pre-transplant immunology		
DSA-positive (n, %)	1	3.3
Recipient CMV-IgG positive (n, %)	17	56
Donor CMV-IgG positive (n, %)	18	60
Transplant-related variables		
First transplant (n, %)	27	90
Second transplant (n, %)	3	10
HLA A/B/DR mismatch sum (median, IQR)	3	2–4
Lymphocyte-depleting agent (n, %)	1	3.3
Desensitization: IA (n, %)	1	3.3
Tacrolimus (n, %)	30	100
MMF/MPA (n, %)	30	100
Steroids	30	100
Time to Double-J removal (days, median, IQR)	56	43–75
BKPyV-associated data		
Time to first VP1-urinary-FC post Tx (months, median, IQR)	10	2–16
Time to peak urinary-VP1-FC post Tx (months, median, IQR)	11.5	2.7–18.1
BKVPyV-DNAuria at first urinary-VP1-FC (c/mL, median, IQR)	3.3 × 10^6^	2.5 × 10^1^–4.0 × 10^9^
BKPyV-DNAemia at first urinary-VP1-FC (c/mL, median, IQR)	4.0 × 10^3^	4.0 × 10^2^–9.0 × 10^3^
BKPyV-DNAemia peak after first urinary-VP1-FC (c/mL, median, IQR)	6.0 × 10^3^	1.0 × 10^3^–2.0 × 10^4^
Patients with JCPyV-DNAemia-only (n/%)	2	6
Patients with combined JCPyV + BKPyV-DNAemia (n/%)	2	6
JCPyV-DNAuria positive (n/%)	8	28
JCPyV-DNAuria (c/mL) (median, IQR)	7.1 × 10^6^	1.1 × 10^6^–2.2 × 10^8^
JCPyV-DNAemia positive (n/%)	4	13
JCPyV-DNAemia (c/mL) (median, IQR)	3.2 × 10^3^	1.0 × 10^2^–8.4 × 10^4^
Tacrolimus trough levels after sampling (ng/mL, median, IQR)		
Month 0 (=baseline urinary-VP1-FC sampling)	7.8	6.9–9.3
Month 1	6.9	5.6–8.9
Month 2	5.9	4.5–9.0
Month 3	6.5	5.4–8.5
Month 4	6.6	5.9–7.1
Month 5	6.7	5.8–8.5
Month 6	6.8	5.7–8.1
Serum creatinine after sampling (mg/dL, median, IQR)		
Month 0 (=baseline urinary-VP1-FC sampling)	1.9	1.1–4.8
Month 1	1.9	1.3–4.3
Month 2	2.0	1.2–3.9
Month 3	2.1	1.1–4.1
Month 4	1.9	1.3–3.4
Month 5	1.9	1.3–3.6
Month 6	2.0	1.3–3.7
MMF/MPA reduction by 50% during episode (n, %)	16	53
MMF/MPA reduction by >50% during episode (n, %)	13	43

Baseline variables of study population:

^a^ Congenital causes of end-stage renal disease (ESRD): agenesis, Alport syndrome, autosomal dominant polycystic kidney disease, autosomal recessive polycystic kidney disease, urethral valve, anatomic malformations.

^b^ Glomerular: FSGS, glomerulonephritis, IgA nephropathy.

^c^ Metabolic/vascular causes: diabetes mellitus, hypertension, amyloidosis, EPH, gestosis; ESRD, end-stage renal disease; DSA, donor-specific antibody; CMV, cytomegalovirus; HLA, human leukocyte antigen; ATG, antithymocyte globulin; IA, immunoadsorption; MMF, mycophenolate mofetil; MPA, mycophenolic acid; BKPyV, BK, polyomavirus; VP1-FC, VP1-flow cytometry; Tx, transplantation; JCPyV, JC, polyomavirus; DNAuria, viral DNA, detected in urine; DNAemia, viral DNA, detected in plasma.

Our control cohort comprises 21 KTX recipients screened during the study, of whom 62% were male, with a median age of 63 years (IQR 50.0–67.5). All had negative BKPyV-DNAemia and no clinically relevant BKPyV-DNAuria (DNA-uria centered at zero; median 0 copies; IQR 0–145) and low JCPyV-DNAuria (median 0 copies; IQR 0–2,850). Correspondingly, FC measurements were low and clustered around baseline, with a median value of 0.0 (IQR 0.0–3.2). In the control cohort 9 (43%) biopsies were performed, 7 (33%) after the VP1-FC sampling. All were negative for BKPyVAN.

### Baseline Cross-Assay Quantification of VP1-Positive Urinary Cells

Urinary-VP1-FC using the rabbit polyclonal anti-VP1 antibody detected a median of 13% (14%–29%, n = 30) VP1-positive urinary epithelial cells. When the same cytospin was examined by indirect immunofluorescence, the rabbit-derived immunoreagent produced a median signal of 10% positive cells (0%–84%, n = 30). In contrast, the subset stained with the mouse monoclonal antibody averaged 6% (2%–40%, n = 19). Classical hematoxylin-eosin morphology identified decoy-like nuclei in 3.5% (0%–40%, n = 30) of cells, and the pathologist’s semi-quantitative decoy cell scoring showed a median of 2% (0%–25%, n = 21) decoy cells. In the BK-negative control group, all baseline cross-assay measurements were centered at background levels, with median values of 0.0 for VP1-FC, indirect immunofluorescence, and hematoxylin–eosin assessment. Supplementary Figure S2 shows the representative confocal immunofluorescence of urine sediment from a 58-year-old male KTX recipient with BKPyVAN, with numerous VP1-positive cells at low magnification. The detailed fluorescence intensity measurements of five representative transplant recipients are provided in Supplementary Figure S3, presenting the technical performance of the urinary VP1 assay.

### Urinary-VP1-Positive Cell Burden and Diagnostic Certainty of BKPyVAN

**FIGURE 2:**
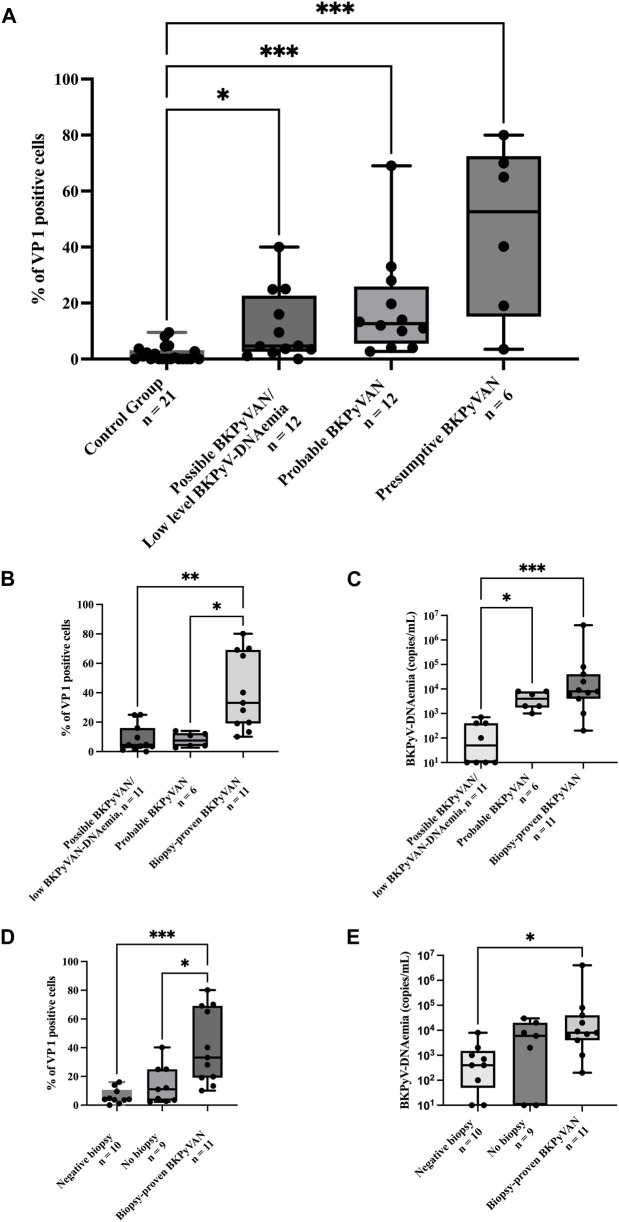
Urinary VP1-positive cell burden and BKPyV-DNAemia by BKPyVAN status; VP1 positivity was calculated as the percentage of VP1-positive cells among 200 DAPI-positive, non-polymorphic nuclei per sample, excluding debris and polymorphonuclear cells; **(A)** Urinary VP1-positive cell percentages in viremic recipients at study inclusion across BKPyV diagnostic certainty categories and the control group (p < 0.001). **(B)** Box-and-whisker plots of urinary VP1-positive cell percentages in patients with possible BKPyVAN with low-level BKPyV DNAemia (n = 11), probable BKPyVAN (n = 6), and biopsy-proven BKPyVAN (n = 11), demonstrating a significant overall group difference (Kruskal–Wallis p < 0.001). Median VP1 positivity increased stepwise across categories [possible: 4.5% (IQR 3.5–14.0), probable: 12.0% (IQR 6.0–22.0), biopsy-proven: 33.0% (IQR19.0–69.0)]. **(C)** Plasma BKPyV DNAemia across the same diagnostic tiers, showing significant differences between groups (Kruskal–Wallis p < 0.001). **(D)** Urinary VP1-positive cell percentages stratified by biopsy status: biopsy-negative (n = 10), no biopsy (n = 9), and biopsy-proven BKPyVAN (n = 11), with significantly higher VP1 burden in biopsy-proven cases (Kruskal–Wallis p = 0.0009). **(E)** Plasma BKPyV DNAemia according to biopsy status, demonstrating significant differences across groups (Kruskal–Wallis p < 0.001).All panels depict baseline measurements obtained at study inclusion.

Among 30 recipients stratified by baseline plasma BKPyV-DNAemia (Figure 1), urinary VP1-positive cell percentages differed significantly across groups (*p* = 0.04). In Dunn’s post-hoc comparisons, recipients with presumptive BKPyVAN showed a greater VP1 burden than those with possible BKPyVAN (adjusted *p* = 0.03). When BK-negative controls were included in the same model, controls showed consistently lower VP1 levels than all BKPyVAN categories (Figure 2A). Among recipients stratified by diagnostic certainty (possible BKPyVAN with low-level BKPyV-DNAemia, probable BKPyVAN, and biopsy-proven BKPyVAN), urinary VP1-positive cell percentages increased progressively across tiers, with a significant overall group difference (Figure 2B; p < 0.001). Plasma BKPyV-DNAemia likewise varied significantly by diagnostic tier (Figure 2C; p < 0.001). Stratification by biopsy status demonstrated significant differences in urinary VP1 burden (Figure 2D; p = 0.0009) and plasma BKPyV-DNAemia (Figure 2E; p < 0.001). Finally, patients with isolated BKPyV-DNAuria had significantly lower urinary VP1 positivity than those with concurrent BKPyV-DNAemia (p = 0.02).

### Temporal Decline in Urinary VP1-Positive Cells and Parallel BKPyV-DNAemia Measurements

**FIGURE 3:**
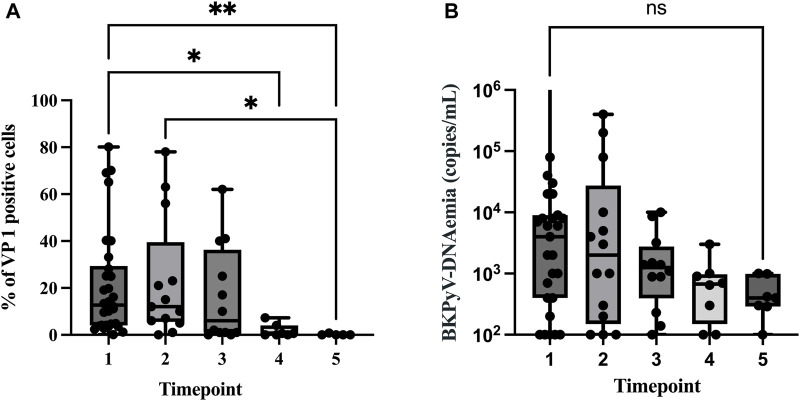
Longitudinal decline in urinary VP1-positive cells and BKPyV-DNAemia. **(A)** Box-and-whisker plots depict the percentage of VP1-positive epithelial cells at each of the five scheduled samplings (Timepoints, TP1-5). Boxes indicate the inter-quartile range (IQR), horizontal bars represent medians. Medians (IQR) fell from 13% (4%–29%) at Timepoint 1 (TP1) to 0% (0%–0.4%) at TP5, illustrating a progressive loss of detectable VP1 antigen. Median timepoints after the study inclusion for the five collections were: baseline (as Timepoint1; TP 1), 21d for TP2, 53d for TP3, 109d for TP4, and 203d for TP5. The corresponding inter-quartile ranges (IQR) were 16–65 days (d), 34-111d, 59-220d and 153-218d. Pairwise Dunn tests (family-wise α = 0.05) identified significant reductions between early and late measurements: TP1 vs. TP4 (p = 0.02*), TP1 vs. TP5 (p = 0.005**), TP2 vs. TP4 (p = 0.05), and TP2 vs. TP5 (p = 0.01). Asterisks above the boxes mark these differences (*<0.05; **<0.01); non-significant comparisons are omitted for clarity. **(B)** Box-and-whisker plots of parallel BKPyV DNAemia measurements (copies/mL) at five sequential urine collections (TP1-TP5).

Across the five flow-cytometric assessments, the distribution of VP1-positive cells became progressively narrower and shifted toward lower values (Figure 3). Collection timepoints (TPs) for the FC samples are described in Figure 3. At baseline (TP1), the median VP1 burden was 13% (IQR 4%–29%). At TP2 the median fell to 11% (IQR 4%–31%), then to 6% (IQR 1%–36%) at TP3, and to 0.6% (IQR 0%–4%) at TP4. At TP5 the median had declined to 0% (IQR 0%–0.4%); *p* < 0.001. Collectively, these data indicate that the bulk of VP1 clearance occurred between months two and four post-urinary-VP1-FC sampling (i.e., between TP3 and TP4), after which the viral signal plateaued to nearly background levels in almost all patients (Figure 3A). Parallel to the flow-cytometric quantification of VP1-positive cells, at TP1, the median viral load was 4.0 × 10^3^ copies/mL (IQR 4.0 × 10^2^–9.0 × 10^3^), which declined to 2.0 × 10^3^ copies/mL (IQR 1.5 × 10^2^–2.8 × 10^4^) at TP2 and to 1.3 × 10^3^ copies/mL (IQR 4.0 × 10^2^–2.9 × 10^3^) at TP3. By TP4 the median had fallen further to 5.0 × 10^2^ copies/mL (IQR 1.3 × 10^2^–1.0 × 10^3^) and at TP5 to 4.0 × 10^2^ copies/mL (IQR 2.0 × 10^2^–1.0 × 10^3^), despite complete loss of VP1 signal by FC at TP5. Figure 3B, shows a rapid initial drop in DNAemia between TP1 and TP2, followed by a gradual plateau towards low-level DNAemia thereafter. Across visits, urinary VP1% showed a positive correlation with BKPyV-DNAemia. At TP1, the correlation was moderate (rho = 0.59, *p* = 0.001), but it was strong at TP2 (rho = 0.75, *p* = 0.01). At later visits, correlations were weaker and not statistically significant.

### ROC Analysis for the Diagnostic Performance of VP1 Liquid Biopsy

**FIGURE 4:**
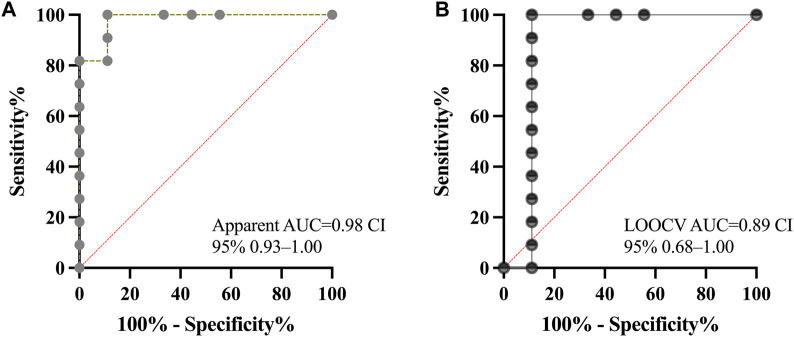
Receiver-operating-characteristic curves for urinary-VP1-FC in detecting biopsy-proven BKPyVAN. **(A)** depicts the apparent ROC obtained when the model was both trained and tested on all 20 recipients with available biopsy after the sampling (13 biopsies from study group and 7 biopsies from the control group; AUC = 0.98). **(B)** shows the leave-one-out cross-validated ROC, in which each patient was predicted by a model that excluded his or her data (AUC = 0.89). Diagonal line = reference for random classification (AUC = 0.50).

Receiver-operating-characteristic analysis for urinary VP1-FC, using biopsy-proven BKPyVAN as the endpoint of interest and restricted to 20 recipients biopsied after study inclusion (including 7 available biopsies from the control group), demonstrated excellent diagnostic performance when using baseline (TP1) values. The AUC was 0.98 (95% CI 0.93–1.00, p = 0.0003; Figure 4A). A VP1 threshold of 7.4% achieved 100% sensitivity and 88.9% specificity. Increasing the cutoff to 11.7% resulted in a sensitivity of 90.9% and specificity of 88.9%, whereas a stricter rule-in threshold of 17.5% yielded sensitivity of 81.8% with 100% specificity, thereby favoring confirmation over detection. In LOOCV, urinary VP1-FC retained good discriminatory performance (AUC 0.89, 95% CI 0.68–1.00; p = 0.0034) despite the small cohort (Figure 4B). However, when separating groups based on possible, probable, and presumptive BKPyVAN using viremia levels at TP1, AUC for the prediction of BKPyVAN by urinary VP1-FC applying a cutoff of 17.5% was 0.74 for possible, 0.52 for probable, and 0.80 for presumptive BKPyVAN. Corresponding values for sensitivity were 47%, 33%, and 83%, and for specificity were 73%, 56%, and 71%. The optimal VP1‐burden thresholds maximizing Youden’s index were >10% for possible BKPyVAN (sensitivity 79%, specificity 73%), >4% for probable BKPyVAN (sensitivity 92%, specificity 33%), and >40% for presumptive BKPyVAN (sensitivity 67%, specificity 96%). The secondary analysis using the combined logistic model of urinary VP1 and BKPyV-DNAemia demonstrated the strongest predictive performance for biopsy-proven BKPyVAN (n = 13, AUC = 0.97). Predicted probability surfaces revealed that probabilities exceeded 0.95 already at approximately 18%–20% VP1 positive cells, even at low viral loads, and approached 0.99 beyond 25% VP1 across any BKPyV-DNAemia (Supplementary Figure S4).

### Longitudinal FC Kinetics

**FIGURE 5:**
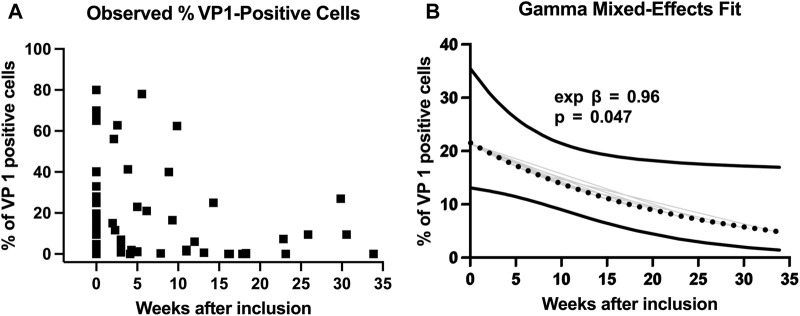
Time course of urinary VP1-positive cells and mixed-effects model fit. **(A)** Observed percentages of VP1-positive epithelial cells measured in 68 urine samples from 30 kidney-transplant recipients. **(B)** The dotted black line shows the population-level trajectory predicted by the gamma-log mixed-effects model, and the area above and below it denotes its 95% confidence interval. Grey lines indicate the individual VP1 trajectories. According to the model, VP1 positivity declines by ≈ 4% per week (expβ = 0.96, p = 0.047), mirroring gradual viral clearance after diagnosis.

A total of 68 urine samples from 30 KTX recipients were available for mixed-effects modelling. At TP1, VP1 load was 21% (geometric mean, 95% CI 13%–35%). Time after diagnosis (start of the reduction of immunosuppression) was an independent predictor for VP1 decline: β = −0.044 ± 0.022, *p* = 0.047. Exponentiation of the coefficient indicated a 4% weekly decline in VP1-positive cells (expβ = 0.96, 95% CI 0.92–1.00). Raw observations are displayed in Figure 5A, while a population-level fitted curve with its 95% CI is shown in Figure 5B. To define a clinically meaningful threshold for VP1 positivity, we reclassified VP1 negativity as ≤1% to avoid trace analytical noise. We then examined VP1-positivity across predefined BKPyV-DNAemia bins. For the BKPyV-DNAemia in a range of 200–700 copies/mL, VP1 was almost always absent, with a median of 0% (IQR 0%–0.95% in 2 × 10^2^–4 × 10^2^; 0%–0.87% in 4 × 10^2^–5 × 10^2^) and positivity observed in only 3 of 12 cases (25%). By contrast, VP1 positivity increased substantially with higher viremia. At 7 × 10^2^–1 × 10^3^ copies/mL, 67% of samples were positive (median 2.65%, IQR 0.6–12.2), and at 1 × 10^3^–3 × 10^3^ copies/mL, 58% were positive (median 3.35%, IQR 0.9–12.9). Above 3 × 10^3^ copies/mL, VP1 positivity was frequent (3 × 10^3^–10 × 10^4^: 86%; 1 × 10^4^–1 × 10^5^: 88%), with median VP1 levels rising steeply (11.3% and 60.6%, respectively). These results indicate that VP1 positivity becomes unlikely once plasma BKPyV DNAemia falls into the ≤500–700 copies/mL range.

## Discussion

In this prospective cohort of KTX recipients, we demonstrate that VP1-positive cell quantification via urinary-VP1-FC might provide a sensitive non-invasive marker to monitor the course of BKPyV infection across different BKPyV-associated endpoints. At baseline, recipients across diagnostic certainty tiers exhibited a stepwise increase in median VP1 burden. Biopsy-proven BKPyVAN revealed the highest VP1 levels, underscoring the relationship between cellular antigen load and disease severity. Our control cohort demonstrated that urinary VP1-detection was largely confined to BKPyV-affected recipients with controls being at assay background-levels only. Moreover, longitudinal sampling revealed a rapid decline in VP1-positive cells between months 2 and 4 post-sampling, paralleling, but ultimately outlasting reductions in plasma BKPyV-DNAemia. Finally, ROC analysis confirmed excellent discriminative performance (AUC 0.98; 95% CI 0.93–1.00) of baseline VP1-FC for BKPyVAN, with an optimal cut-off at 11.6% VP1-positive cells yielding both high sensitivity and specificity. Our preliminary findings support further validation of urinary VP1-FC trajectories for monitoring the course of BKPyV infection. Our results align with earlier reports that direct enumeration of infected cells in conjunction with qPCR may more accurately reflect viral injury [27–29]. Traditional decoy-cell scoring is limited by subjective interpretation and low throughput as well as the requirement for trained pathologists [30]. Immunofluorescence assays targeting VP1 may improve specificity when compared to urinary diagnostic methods [27, 31]. Our urinary VP1-FC assay builds on the availability of standard flow cytometers in routine laboratories. Once established, it may deliver rapid (<24 h), quantitative high-throughput detection of infected reno-urinary epithelial cells, overcoming logistical and technical constraints of other biomarkers for BKPyVAN [23]. The *Haufen test*, which detects viral aggregates in urine by electron microscopy, has shown good diagnostic accuracy in independent cohorts. However, its reliance on electron microscopy and labor-intensive protocols has limited its clinical adoption [23, 32]. Similarly, early transcriptomic assays measuring urinary VP1, granzyme B, and PI-9 mRNA reported high sensitivity and specificity for biopsy-proven BKPyVAN, but these methods were technically demanding, rarely reproduced, and did not address viral clearance kinetics [33]. In contrast, urinary VP1-FC provides direct quantification of infected epithelial cells and may offer the advantage to observe viral clearance in advance to the resolution of DNAemia. This may aid in the evaluation of success or failure during/after reduction of immunosuppression and its potential re-installation.

Temporal kinetics of VP1-positivity mirror the clinical course of BKPyVAN. At baseline we observed a median burden of 13% VP1-positive cells, which declined to 0% by the fifth sampling (≈7 months after study inclusion). Notably, the steepest drop occurred after the second month, coinciding with immunosuppression reduction. Additionally, our threshold analysis demonstrated that urinary VP1-FC is negative in low range viremia, whereas positivity is frequently observed above 10^3^ c/mL. Persisting viremia with or without further graft damage due to BKPyV has already been reported [34–37]. Those findings show a possible dissociation between the clearance of cell‐associated antigen and the persistence of circulating viral genomes. Such compartmentalized kinetics suggest that urinary VP1-FC may report on the resolution of intrarenal infection, capturing the disappearance of actively replicating epithelial reservoirs, whereas PCR-based DNAemia measurements can remain elevated owing to free virions, non-cell-associated viral debris, concentration, and genomic variation or mutations in primers [38–40]. Therefore, in scenarios where DNAemia plateaus, reliance on PCR alone may underestimate viral clearance on the tissue-level, underscoring the added value of our approach for guiding immunosuppression (re)-adjustments. ROC‐derived thresholds for urinary VP1-FC compared favorably with those reported for urine PCR and decoy‐cell counts. An AUC of 0.98 for biopsy-proven BKPyVAN is even higher than previously reported in small-series estimates for urinary cell assays (AUC≈0.85–0.92) and exceeds typical performance of urinary viral load cut-offs alone (AUC≈0.75–0.85) [27, 31, 41, 42]. The 11% VP1 burden threshold optimizes Youden’s index, yet a lower cut-off of 7% captures all cases at the expense of specificity. This trade-off mirrors clinical dilemmas in BKPyV management, where early detection must be balanced against overtreatment risks [43]. Additionally, the absence of BKPyVAN in early biopsies indicates that preceding histological findings did not bias VP1 measurements via changes in immunosuppressive therapy. However, while ROC analyses suggested excellent discrimination, the low sample size increases the risk of optimistic performance estimates despite LOOCV, and confidence intervals remain wide. Accordingly, these results should be interpreted with caution and only as hypothesis-generating and require confirmation in larger, independent cohorts. Future work will focus on assay standardization (pre-analytical handling, gating strategy, and inter-run calibration) and prospective multicenter validation with pre-specified thresholds and quality-control procedures.

Mixed-effects modeling quantified a weekly 4% decline in VP1 positivity post-diagnosis, reinforcing the assay’s potential for dynamic monitoring. When combined with cross-validated discrimination, the joint probability modeling with BKPyV-DNAemia confirmed that urinary VP1-positivity >20% already corresponds to >95% predicted probability of biopsy-proven BKPyVAN. Together, these kinetics support further exploration with respect to biopsy-sparing approaches and non-invasive monitoring of therapeutic re-escalation of immunosuppression.

Despite these promising findings, several limitations merit discussion. First, 30 recipients from a single center limit generalizability and may introduce selection bias. Second, 30% of patients were not biopsied, potentially misclassifying some as BKPyVAN-negative; although non-invasive assays may obviate biopsy in many cases, such missing histology reduces certainty in diagnostic accuracy estimates. Additionally, the observed heterogeneity in biopsy timing relative to VP1-FC sampling reflects routine clinical workflows and supports interpretation of VP1-FC as a dynamic marker of polyomavirus activity rather than a biopsy-synchronized diagnostic test. While we included a virologically negative, stable transplant control cohort, this group does not capture all inflammatory or ischemic graft pathologies that may confound urinary cellular readouts. Consequently, the reported specificity and AUC primarily reflect performance within a BKPyV-reactivation framework and should not be interpreted as real-world specificity across all causes of graft dysfunction. Larger validation studies incorporating relevant differential diagnoses are required prior to clinical implementation. Further, the flow-cytometric assay did not include epithelial lineage markers (e.g., pan-cytokeratin or EpCAM); although urinary samples in BKPyV infection are predominantly epithelial, inflammatory cell admixture in injured grafts cannot be excluded, and future studies should confirm the cellular origin of VP1-positive events. Another limitation is that the polyclonal anti-VP1 antibody recognizes conserved VP1 epitopes across polyomaviruses, including BKPyV and JCPyV. While this precludes viral species discrimination at the cellular level, it aligns with routine diagnostic practice, where SV40 immunohistochemistry similarly detects polyomavirus infection without species specificity. Consequently, the assay should be interpreted as reflecting active polyomavirus replication rather than BKPyV-exclusive disease. In addition, as demonstrated by other research groups, BKPyV frequently acquires mutations - particularly in the VP1 protein - potentially leading to false negative results in our assay [44, 45]. Lastly, urine samples with more than 50% granulocytes were excluded because high granulocyte content interferes with reliable flow-cytometric acquisition and VP1 signal interpretation. This exclusion may introduce selection bias, as granulocyturia can occur in the context of concurrent infection or acute rejection, conditions that may overlap clinically with BKPyVAN. However, this limitation reflects a technical constraint of the current assay rather than *post hoc* selection, and the study population remains representative of patients in whom non-invasive urinary monitoring is clinically applicable.

Strengths of the study include the current application of the hierarchical BKPyVAN diagnostic algorithm, parallel comparison of urinary VP1-FC with multiple detection modalities (PCR, IFL, decoy cells), and robust statistical methods to avoid overfitting. Moreover, the direct measurement of VP1 on exfoliated cells target the pathogen at its site of action, arguably offering greater pathophysiological relevance than surrogate markers.

If future studies confirm our findings, integration of urinary VP1-FC into post-transplant surveillance may complement current BKPyV monitoring strategies. This proof-of-concept cohort lacks a fully representative control population and the observed diagnostic performance requires validation in larger cohorts before conclusions regarding real-world specificity or clinical utility can be drawn. Standardization of assay protocols and interlaboratory calibration will be essential for broader implementation. While PCR remains the gold standard for detecting BKPyV replication, VP1-FC directly quantifies cell-associated viral antigen in exfoliated tubular epithelial cells and may provide complementary insight into intrarenal infection dynamics, particularly in the setting of persistent low-level or plateauing DNAemia. The potential role of urinary VP1-FC in informing immunosuppression management, including re-escalation strategies after apparent cellular viral clearance, should therefore be evaluated in future prospective studies.

## Data Availability

The raw data supporting the conclusions of this article will be made available by the authors, without undue reservation.
