# Supplementary material for: Urinary VP1 Flow Cytometry as a Complementary Approach for BK Polyomavirus Monitoring: A Proof-Of-Concept Study
Source: Transpl Int. 2026 Apr 8;39:15780. doi: 10.3389/ti.2026.15780 (PMC13099444; doi:10.3389/ti.2026.15780)
Supplement: Supplementary file 1 [file Supplementaryfile1.docx]

**Supplemental Materials**

**Supplemental Methods**

**Definition of BKPyV-associated endpoints**

BKPyVAN was categorized according to the most recent international consensus framework into four grades: possible, probable, presumptive, and definite^1^. Possible BKPyVAN was assigned when there was evidence of substantial BKPyV-DNAuria [BKPyV DNA in urine >10⁷ copies/m (c/mL)] or decoy cells in urine sediment (with additional low level BKPyV-DNAemia defined as <10^3^ c/mL); probable BKPyVAN required BKPyV-DNAemia>10³ c/mL sustained for at least two weeks (i.e., on two consecutive measurements within a three-week window); presumptive BKPyVAN was defined by BKPyV-DNAemia >10⁴ c/mL on ≥1 measurement and definite (i.e. biopsy-proven) BKPyVAN demanded histopathological confirmation on graft biopsy demonstrating viral cytopathic changes with positive SV40. Biopsies were assessed for adequacy according to institutional standards. The minimum acceptable sample was defined as 7 glomeruli and 1 artery obtained from at least two distinct cores ^2^.

## **Test methods for urine and plasma BKPyV**

Urinary and plasma BKPyV and JCPyV-DNAemia was quantified as part of routine post-transplant polyomavirus surveillance at our center in all kidney transplant recipients included in the study, following international screening recommendations, acknowledging that polyomavirus nephropathy may rarely be caused by JCPyV. Viral loads were quantified according to the manufacturer’s protocol using the GeneProof BK and JC Virus PCR Kit-IVDR (GeneProof, Brno, Czech Republic) as part of our center’s routine procedures. The applied method of BKPyV-DNAemia quantification, and the institution’s screening protocol have been described previously; assay’s lower limit of quantification (LLOQ) of 100 copies/mL, limit of detection (LOD): 70 copies/mL ^3,4^. Each patient provided up to five urine samples, which were collected during sequential routine follow-up visits as part of standard post-transplant care. As the study was embedded in clinical routine, no additional visits were scheduled. For analytical purposes, samples were labeled as timepoints (TP): TP1 (baseline) through TP5, reflecting visit order rather than fixed time intervals after transplantation.

## **Flow-cytometric analysis of VP1-positive urinary epithelial cells**

Approximately 30 mL of first-morning urine was processed immediately after collection; samples were transported directly to the laboratory, centrifuged at 2,000 g for 10 minutes to obtain a cell pellet, and the pellet was subsequently suspended in tissue culture medium and ethanol-fixed (RPMI 1640 with 2% FCS and ampicillin-streptomycin antibiotics), typically within 30 minutes of urine collection (predefined maximum acceptable time-to-fixation of 150 minutes was applied). This standardized pre-analytical workflow was used to minimize cellular autolysis and antigen degradation. Aliquots of this cell suspension were subjected to further centrifugation at 1500 RPM. The resulting cell pellet was treated with 2500 µl of 70% ethanol and allowed to incubate for 60 minutes to fix the cells. Subsequently, the fixed cells were washed with 3 ml of PBS and centrifuged at 2500 RPM for 3 minutes. The supernatant was discarded, and the cell pellet was incubated in 1000 µl of antibody staining solution, containing rabbit anti-VP1 antibody at a dilution of 1:1000 in PBS, or a control antibody over night at 4°C. After one wash with 3 ml of PBS, the cell suspension was centrifuged again at 2500 RPM for 3 minutes. The supernatant was discarded, and the cell pellet was incubated with Alexa Fluor 488-labeled goat anti-rabbit secondary antibody for 60 minutes, with frequent tapping of the tube. Following two washes with PBS, the cell pellet was suspended in 500 µl of PBS containing DAPI and analyzed using a FACSCanto^TM^. Urine samples were collected at up to five TP per patient. FC data were processed using custom Python scripts executed within JupyterLab (Project Jupyter, 2024), accessed via the Anaconda Navigator distribution (Anaconda Inc., 2024). Samples with massive granulocyturia were deemed technical failures and excluded from urinary-VP1-FC analysis, as high granulocyte counts compromise accurate VP1 quantification. Because all urinary cells were fixed and permeabilized prior to staining, viability testing was not applicable, and FSC/SSC plots were not informative beyond debris exclusion due to altered refractive properties of the fixed cells. VP1 positivity was quantified by immunofluorescence analysis using a standardized, cell-count–based approach. For each sample, 200 DAPI-positive, non-polymorphic nuclei were counted and defined as the denominator (100%). Polymorphonuclear granulocytes, non-cellular events, and debris were excluded prior to quantification. VP1 positivity was calculated as the percentage of VP1-positive nuclei among these 200 DAPI-positive cells.

Classical doublet discrimination was not applied, as enlarged, tetraploid, and polyploid nuclei represent known cytopathic features of polyomavirus infection and were therefore intentionally retained in the analysis.

## **Cytopreparations**

Urine sediment cells were maintained in culture medium as described above (RPMI 1640 supplemented with 2% FCS and ampicillin-streptomycin). A 60 μl aliquot was applied to the funnel of a cytocentrifuge and centrifuged at 1200 rpm for 3 minutes. The resulting cytopreparation was air-dried for 1 hour, followed by fixation in acetone for immunostaining or in the fixation solution of the Hemacolor^Ⓡ^ (Merck, Merck KGaA, Darmstadt, Germany; Millipore, SIG 1116740001) three-component kit for 4 minutes.

## **Immunofluorescence of urinary cells on cytopreparations**

Cytopreparations as described above were air-dried for at least one hour and subsequently fixed in acetone for 5 minutes. A hydrophobic barrier was drawn around the cell-containing area of the slide, which was then pre-wetted with PBS prior to the addition of the primary antibody. The BKPyV VP1-specific mouse monoclonal antibody (4942 Invitrogen, Carlsbad, CA, USA) was diluted 1:60 in PBS and applied to the slide. In parallel the monospecific rabbit anti VP1 was diluted 1:1000 in PBS and applied on a separate cytopreparation. Slides were incubated either overnight at 4 °C or for 2 hours at room temperature, followed by washing in PBS for 10 minutes under constant stirring. A secondary antibody Alexa Fluor 594-conjugated goat anti-mouse IgG (A11032, Invitrogen) or Alexa Fluor 488 goat anti rabbit (A11008, Invitrogen) (diluted 1:700 in PBS) was then applied and incubated for 1 hour at room temperature. DAPI was included in the staining solution for nuclear counterstaining. After a final 10-minute PBS wash under constant gentle stirring, the slides were mounted using 10 µL of Vectashield mounting medium and covered with a glass coverslip. Fluorescence imaging was performed using a Zeiss inverted confocal microscope (Carl Zeiss AG, Oberkochen, Germany) and final image processing was carried out using Adobe Photoshop (version 26 for Mac, San Jose, CA, USA).

## **Decoy cell staining**

One cytopreparation was sequentially stained with eosin, followed by hematoxylin and a washing buffer, using the Hemacolor^Ⓡ^ staining kit (Merck, Merck KGaA, Darmstadt, Germany; Millipore, SIG 1116740001). Cell enumeration was performed via light microscopy. Criteria for identifying decoy cells included a disproportionately high nuclear-to-cytoplasmic ratio, a ground-glass or smoky nuclear appearance with basophilic or amphophilic intranuclear inclusions, and cytoplasm that, when present, exhibited a characteristic tail-like extension.

## **Statistical Analysis**

## Data were analyzed using both descriptive and inferential statistical methods. Continuous variables were assessed for normality with the Shapiro-Wilk test and are presented as means±standard deviations (SD) when normally distributed or as medians with interquartile ranges (IQR) for skewed data. Categorical variables are summarized as frequencies and percentages. Group comparisons were performed using the student’s t-test for normally distributed continuous data or the Mann-Whitney U test for nonparametric data. Kruskal-Wallis’s test was used to compare the differences in BKPyV-DNAemia and urinary-VP1-FC counts in more than two groups. Urinary-VP1-FC data were recorded as percentages, and the intervals between sample collections were documented for assessment of changes over time. The diagnostic performance of the urinary-VP1-FC assays was evaluated by constructing receiver operating characteristic (ROC) curves, and the area under the curve (AUC) was calculated to determine sensitivity, specificity, positive predictive value, and negative predictive value for BKPyV diagnostic tiers. Preliminary AUC estimates were validated using leave-one-out cross-validation (LOOCV) to assess the robustness of our classification performance. The urinary-VP1-FC measurements were modeled using a generalized linear mixed-effects model (GLMM) with a Gamma distribution and a log link using “glmmTMB” package. The model included time as a fixed effect and a random intercept for each subject to account for the repeated-measures nature of the data. Statistical analyses were conducted using appropriate software packages (GraphPad Prism 10.0.3 (217) Macintosh Version by Software MacKiev, GraphPad Software, LLC, Boston, MA, USA; R version 2024.09.1+394 (2024.09.1+394) by Posit Software, PBC, Boston, MA, USA and IBM SPSS Statistics; version 29.0.2.0.for Mac, Armonk, NY, USA). All statistical tests were two-tailed with a significance level set at *p*<0.05.

**Supplemental Figures**

**
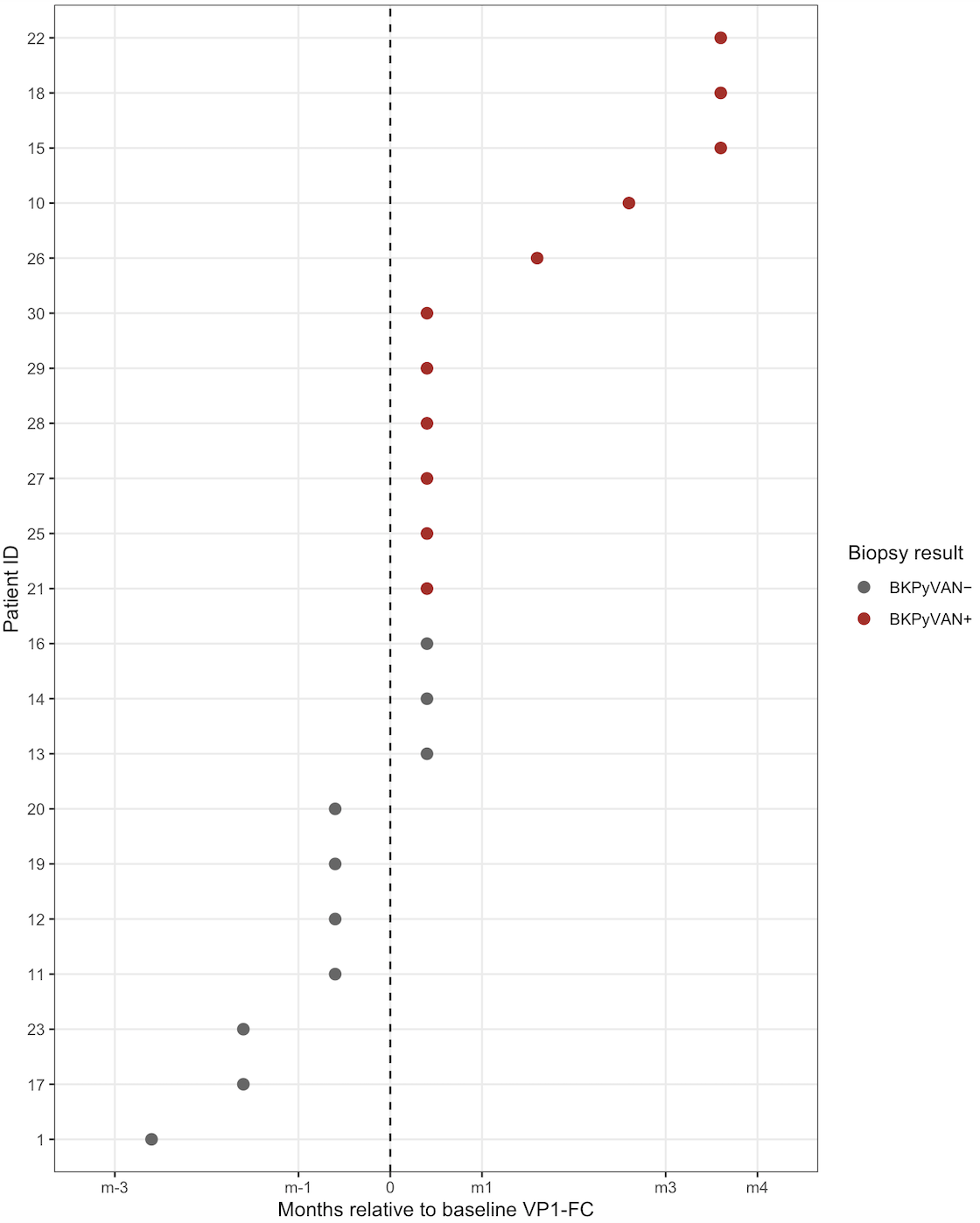
**

**Supplemental Figure S1.** Timeline of 21 biopsies relative to baseline VP-FC for the study group.


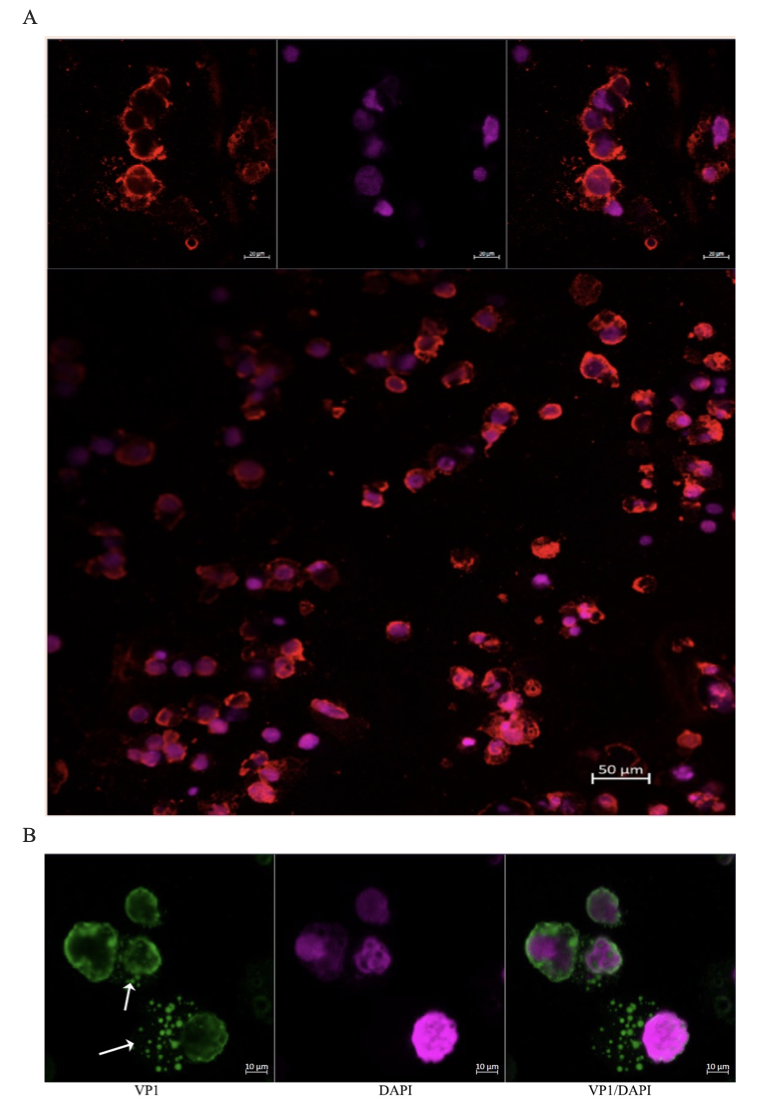


**Supplemental Figure S2.** Panel A) Confocal microscopic validation of the urinary VP1 assay. Urine sediment from a 58‑year‑old male kidney‑allograft recipient, 10 months post‑transplant, was stained with mouse monoclonal anti‑VP1 antibody (red), while nuclei were stained by DAPI (purple). The upper image shows the high‑magnification view of a representative slide demonstrating marked nuclear enlargement, chromatin fragmentation, and loss of cytoplasmic integrity, consistent with BKPyVAN. The lower part shows the low‑magnification overview revealing numerous VP1‑positive cells (red), indicating a high intraluminal viral burden. Scale bar: 20µm, and 50µm. Panel B) Cells originated from a patient with over 60% decoy cells revealing VP1 expression patterns. Cyto-preparations were fixed in acetone and stained with a rabbit anti-VP1 antibody (green), with nuclear counterstaining using DAPI. VP1 signal intensity and localization varied among individual decoy cells, which exhibited a markedly increased nuclear-to-cytoplasmic ratio. Notably, VP1 staining was observed within cytoplasmic vacuoles (arrow), likely filled with viral particles. Scale bar: 10µ


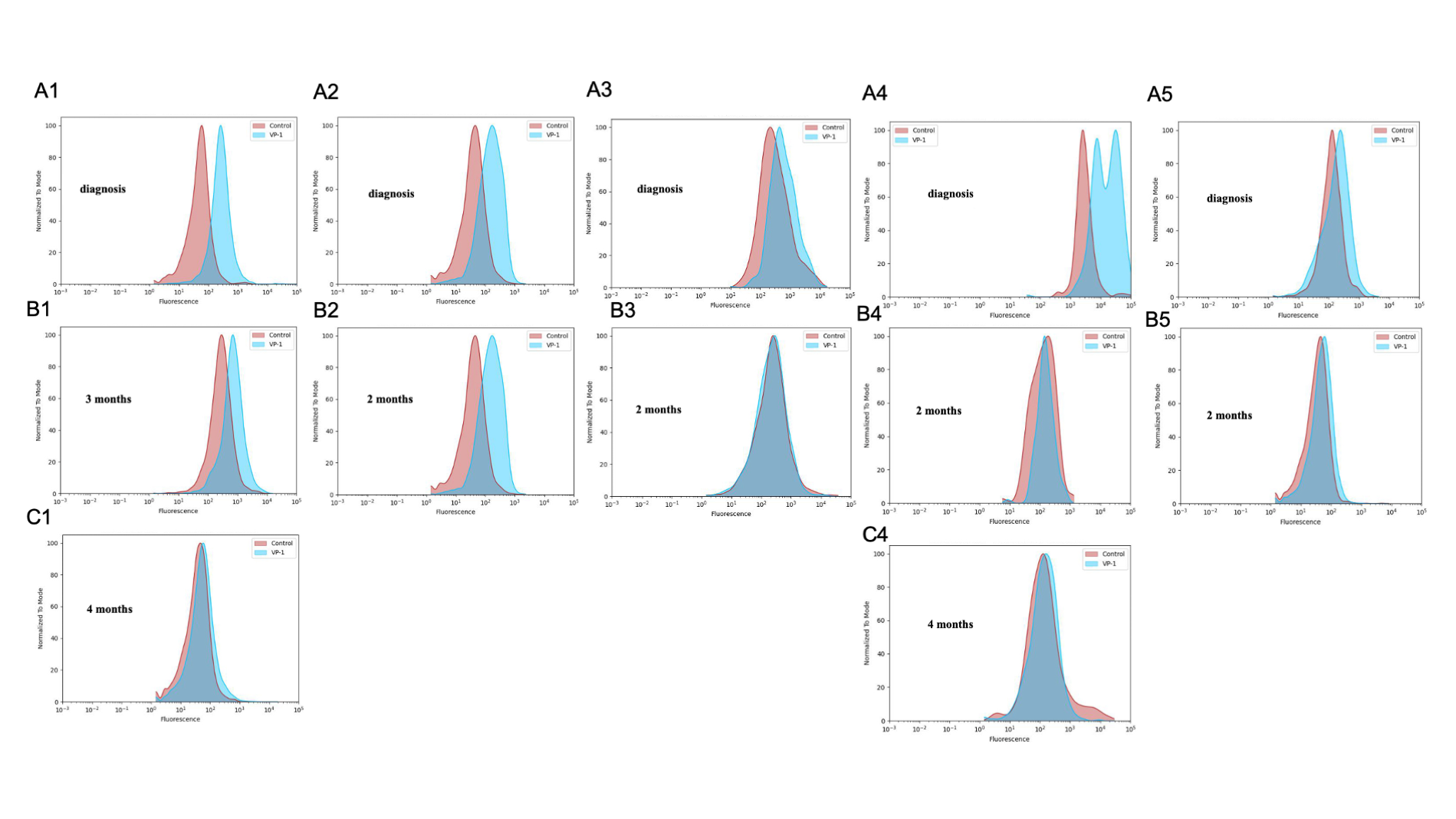


**Supplemental Figure S3**. Flow cytometric analysis of VP1-positive urinary cells from five kidney transplant recipient with BKPyVAN. Panels A1-A5 show initial analysis, where a significant proportion of urinary cells exhibited nuclear enlargement and were positive for VP1 expression (blue). Background fluorescence was assessed using control rabbit serum staining of the same cell population (red). (B) At different timeframes during follow-up, following modification of the immunosuppressive regimen, repeat FC analysis was performed. Panels B1-B5: Following the modification of the immunosuppressive therapy, VP1-positive cells in the urine sediment were markedly reduced. Panels B1-B5, C1+C4: Upon reanalysis at months two, three and four after the initial FC, in most cases VP1-positive staining had decreased in intensity, accompanied by a reduction in the number of positive cells following modification of immunosuppression.

**
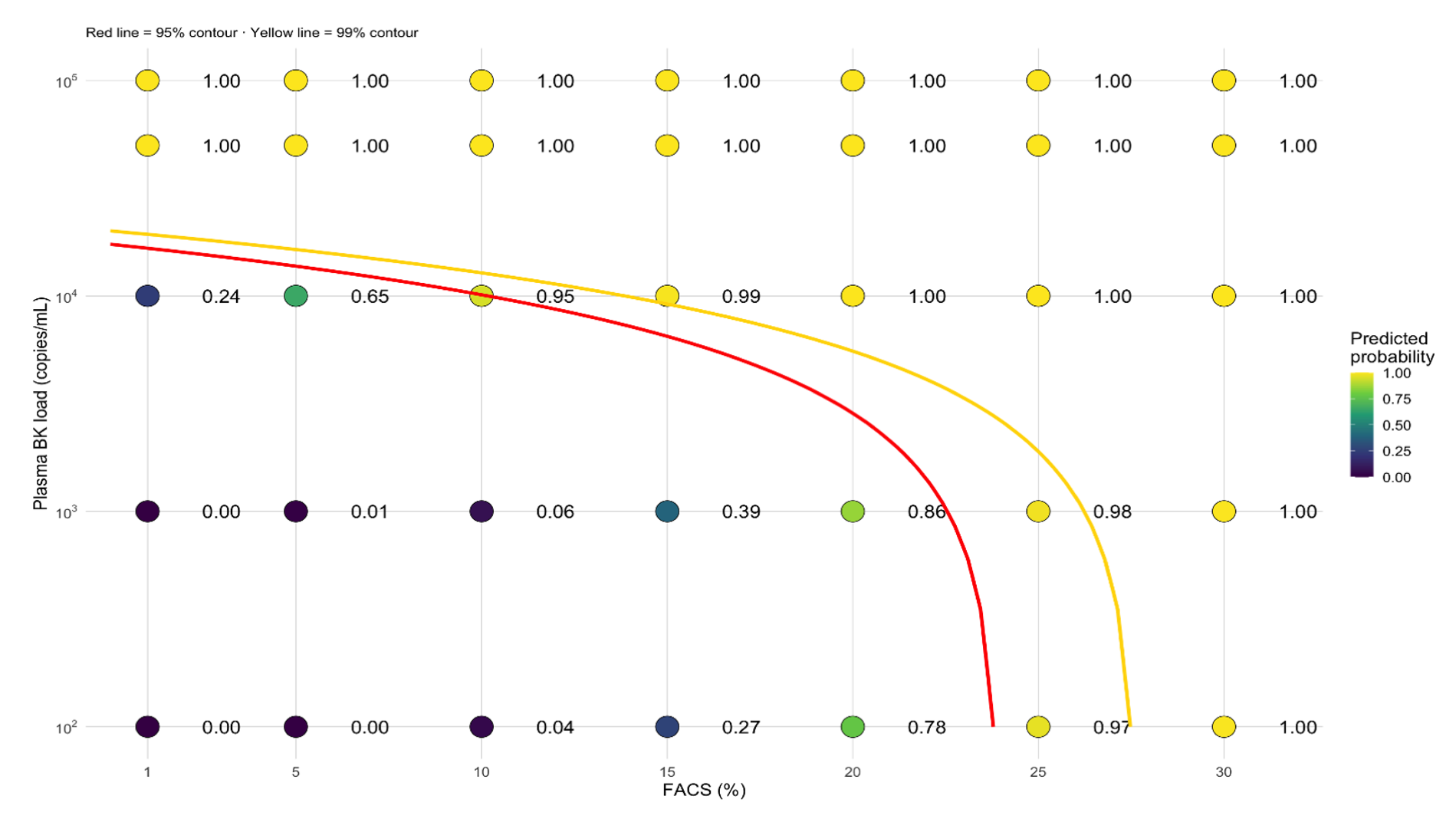
**

**Supplemental Figure S4.** Predicted probability surface for BKPyVAN based on urinary FC-VP1 and BKPyV-DNAemia. The combined logistic regression model integrating urinary VP1-positive epithelial cell percentage and BKPyV-DNAemia predicts the probability of biopsy-proven BKPyVAN (AUC=0.97). Colored circles indicate predicted probabilities for measured combinations of FC-VP1 % and BKPyV-DNAemia, while red and yellow contour lines represent 95 % and 99 % probability thresholds, respectively. Probabilities exceeded 0.95 at approximately 18–20 % VP1 positive cells, even at low plasma BK levels, highlighting the strong predictive value of the urinary “liquid biopsy” signal.

**Supplemental Tables**

**Supplemental Table S1. Number of evaluable samples for tacrolimus and serum creatinine.**

| Timepoint | Tacrolimus trough levels (N) | Serum creatinine (N) |
| --- | --- | --- |
| M0 | 29 | 30 |
| M1 | 25 | 30 |
| M2 | 26 | 29 |
| M3 | 27 | 29 |
| M4 | 26 | 29 |
| M5 | 25 | 27 |
| M6 | 20 | 26 |

**References**

1. Imlay H, Baum P, Brennan DC, et al. Consensus Definitions of BK Polyomavirus Nephropathy in Renal Transplant Recipients for Clinical Trials. *Clin Infect Dis*. Sep 30 2022;75(7):1210-1216. doi:10.1093/cid/ciac071

2. Omić H, Eder M, Schrag TA, et al. Peritubular and Tubulointerstitial Inflammation as Predictors of Impaired Viral Clearance in Polyomavirus Nephropathy. *Journal of Clinical Medicine*. 2024;13(19):5714.

3. Weseslindtner L, Hedman L, Wang Y, et al. Longitudinal assessment of the CXCL10 blood and urine concentration in kidney transplant recipients with BK polyomavirus replication-a retrospective study. Research Support, Non-U.S. Gov't. *Transplant International*. 33(5):555-566.

4. Eder M, Schrag TA, Havel EF, et al. Polyomavirus Nephropathy in ABO Blood Group-Incompatible Kidney Transplantation: Torque Teno Virus and Immunosuppressive Burden as an Approximation to the Problem. *Kidney Int Rep*. Jun 2024;9(6):1730-1741. doi:10.1016/j.ekir.2024.04.003
